# Editorial: Decompressive craniectomy and cranioplasty: challenges and chances volume II

**DOI:** 10.3389/fsurg.2024.1508524

**Published:** 2024-10-25

**Authors:** Julius Höhne, Christian Henker, Ciaran Scott Hill

**Affiliations:** ^1^ Department of Neurosurgery, Paracelsus Medical University, Nuremberg, Germany; ^2^ Department of Neurosurgery, University Hospital Rostock, Rostock, Germany; ^3^ National Hospital for Neurology and Neurosurgery (NHNN), London, United Kingdom

**Keywords:** cranioplasty, decompression, neurosurgery, trauma, stroke, complication, traumatic brain injury, aging population

**Editorial on the Research Topic**
Decompressive craniectomy and cranioplasty: challenges and chances volume II

Decompressive craniectomy (DC) and subsequent cranioplasty (CP) remain cornerstone interventions in the management of life-threatening intracranial hypertension, particularly in cases of traumatic brain injury (TBI) and large ischemic strokes. This Research Topic compiles cutting-edge studies that explore both the immediate benefits and long-term implications of these procedures. The articles presented not only reaffirm the clinical significance of DC and CP but also introduce valuable new insights that broaden our understanding of factors influencing outcomes and complication rates across diverse patient populations.

The aging population introduces new complexities in the management of DC and CP, as older adults often experience outcomes that differ markedly from those of younger patients. Kapapa et al. provide a comprehensive evaluation of DC in patients over 65 years of age, finding that, despite advances in critical and rehabilitative care, older patients continue to face higher mortality and morbidity rates. This trend is compounded by the increasing gap between biological and chronological age—a factor that clinicians must consider when planning DC. The authors advocate for a shift in surgical decision-making that emphasizes the patient's biological age and overall physiological resilience over chronological age alone. Their findings highlight the importance of personalized surgical risk assessments, especially as society continues to age, making it crucial to consider not only immediate postoperative survival but also long-term functional independence and quality of life (Kapapa et al.).

The optimal timing of cranioplasty remains a subject of considerable interest. Early cranioplasty, defined as CP performed within three months of DC, has gained attention due to its potential benefits in neurological recovery and prevention of complications such as the syndrome of the trephined. Yan et al. contribute a significant study offering a predictive model that includes factors such as age, Glasgow Coma Scale (GCS) score, defect area, and the interval between DC and CP. This model provides clinicians with a valuable tool for assessing the likelihood of favorable outcomes following early CP, promoting a more individualized approach to surgical timing. Importantly, their study underscores that early intervention can be performed safely without necessarily increasing the risk of complications, challenging previous assumptions about the risks associated with early CP. By offering a robust framework to predict outcomes, this model has the potential to transform CP planning and patient counseling by aligning surgical timing with patient-specific characteristics (Yan et al.).

Further expanding on the patient-specific approach to DC and CP outcomes, Oliveira et al. explore the impact of morphometric and cerebral radiodensity changes on neurological prognosis in patients with skull defects. Their study demonstrates that parameters such as cortical surface alterations and midline shift reduction correlate significantly with postoperative cognitive outcomes, as measured by tools like the Mini-Mental State Examination (MMSE). This investigation provides a promising avenue for utilizing non-invasive imaging markers to predict postoperative cognitive improvement and recovery potential in CP patients. The results encourage a more nuanced, radiologically guided assessment of skull defect repairs, offering practical methodologies for clinicians to anticipate recovery trajectories and possibly mitigate complications associated with delayed or poorly planned CP (Oliveira et al.).

Complications remain a significant consideration in DC and CP, with post-traumatic hydrocephalus (PTH) being one of the most common and challenging conditions that can arise postoperatively. In this issue, Șerban et al. focus on PTH as a frequent sequela following DC, highlighting the correlation between prolonged mechanical ventilation, extended vasopressor use, and increased PTH risk. Their findings suggest that minimizing the duration of these interventions could potentially lower the incidence of PTH—an insight that is particularly pertinent in intensive care settings where prolonged sedation and mechanical support are often essential. The study raises critical questions about perioperative management and calls for further exploration into optimizing ventilatory support protocols to balance intracranial pressure control with the risk of developing PTH. The implications extend beyond DC, prompting a re-evaluation of critical care practices in neurotrauma to improve long-term outcomes (Șerban et al.).

Collectively, these contributions underscore the multifactorial nature of DC and CP outcomes, emphasizing the importance of a holistic approach that considers patient demographics, surgical timing, perioperative management, and individualized predictive modeling. The studies advocate for a tailored approach to neurosurgical interventions, moving beyond a one-size-fits-all framework. They highlight the need for dynamic decision-making processes that incorporate patient-specific data, enabling neurosurgeons to optimize interventions and improve recovery trajectories.

In summary, this Research Topic offers a comprehensive overview of recent advancements in DC and CP. By addressing the challenges associated with an aging population, refining predictive models for surgical outcomes, leveraging radiological assessments for improved planning, and investigating perioperative factors to minimize complications, these studies contribute significantly to the evolution of these critical neurosurgical procedures. We extend our sincere gratitude to the authors for their rigorous research and to the reviewers whose contributions ensured the quality and relevance of this body of work. This collection serves as a testament to the progress in decompressive surgery and a roadmap for future research aimed at enhancing the safety, efficacy, and personalization of neurosurgical interventions.

